# Performance of Papanicolaou Testing and Detection of Cervical Carcinoma *In Situ* in Participants of Organized Cervical Cancer Screening in South Korea

**DOI:** 10.1371/journal.pone.0035469

**Published:** 2012-04-16

**Authors:** Mi Ah Han, Kui Son Choi, Hoo-Yeon Lee, Jae Kwan Jun, Kyu Won Jung, Sokbom Kang, Eun-Cheol Park

**Affiliations:** 1 Department of Preventive Medicine, College of Medicine, Chosun University, Gwangju, Korea; 2 National Cancer Control Research Institute, National Cancer Center, Goyang, Korea; 3 Department of Social Medicine, Dankook University, College of Medicine, Cheonan, Korea; 4 Branch of Uterine Cancer, Research Institute and Hospital, National Cancer Center, Goyang, Korea; 5 Department of Preventive Medicine and Institute of Health Services Research, Yonsei University, College of Medicine, Seoul, Korea; University of Campinas, Brazil

## Abstract

**Background:**

The present study measured the performance of the Papanicolaou (Pap) test and detection of cervical carcinoma *in situ* (CIS) and cancer in participants of organized cervical cancer screening in South Korea, and examined differences in the proportion of CIS according to socio-demographic factors.

**Methods:**

Data were obtained from the National Cancer Screening Program and National Health Insurance Cancer Screening Program databases. We analyzed data from 4,072,997 screenings of women aged 30 years or older who underwent cervical cancer screening by Pap test between January 1, 2005 and December 31, 2006. We calculated the performances of the Pap test and compared that according to socio-demographic factors.

**Results:**

The positivity rate for all screenings was 6.6%. The cancer detection rate (CDR) and interval cancer rate (ICR) were 0.32 per 1,000 screenings, and 0.13 per 1,000 negative screenings, respectively. About 63.4% of screen-detected CIS+ cases (CIS or invasive cervical cancer) were CIS. The CDR and ICR, and percentage of CIS among all CIS+ were significantly different by age group and health insurance status. The odds ratios of CDR and ICR were higher for Medical Aid Program (MAP) recipients compared with National Health Insurance (NHI) beneficiaries. The likelihood of a detected CIS+ case to be CIS was significantly lower among MAP recipients than among NHI beneficiaries.

**Conclusions:**

The difference in performance of cervical cancer screening among different socio-demographic groups may indicate an important influence of socio-demographic factors on preventive behavior. The findings of the study support the critical need for increasing efforts to raise awareness and provide more screening in at-risk populations, specifically low-income groups.

## Introduction

Despite decreasing cervical cancer incidence and mortality rates in developed countries, cervical cancer remains the third most commonly diagnosed cancer and the third most common cause of cancer mortality among women worldwide [1]. In South Korea, the age-standardized incidence rate of cervical cancer in 2007 was 10.7 per 100,000 women, and cervical cancer was the seventh most common cancer among women. However, the average annual percentage change for 1999–2007 was −4.8%, which shows that cervical cancer is continuously decreasing in Korea [2]. Despite decreasing cervical cancer incidence, the incidence of cervical carcinoma *in situ* (CIS), a precursor of cervical cancer, is continuously increasing. The age-standardized incidence rate of CIS was 8.6 per 100,000 in 1993 and 12.4 per 100,000 in 2002 [3]. Furthermore, the percentage of CIS among all cervical malignancy has increased from 28.5% in 1993 to 40.8% in 2002 [4]. The decreasing trend in cervical cancer incidence and the increasing trend in CIS incidence could largely be due to the introduction of cervical cancer screening in South Korea in the late 1980s. Indeed, the increase in CIS incidence parallels an increase in the use of the Papanicolaou (Pap) test for cervical cancer screening, which has made the detection of CIS much more likely than in the past.

Although cervical cancer incidence and mortality rates are decreasing, the worldwide issue of unequal incidence and mortality by socioeconomic status remains. In many countries, it has been reported that lower income groups have higher cervical cancer incidence and mortality than higher income groups [5], [6], [7], [8]. In Korea, Kim et al. [9] showed that cervical cancer incidence and mortality are unfavorably unequal in lower income groups. Indeed, the benefits of cervical cancer screening and resulting early detection are not shared by all segments of the population. It has been reported that high poverty levels are related to low screening rates [10], [11], and low-income and minority women tend to be diagnosed at later stages and have higher mortality rates [12], [13], [14], [15], [16], [17], [18], [19]. Also, the well-known risk factors of cervical cancer such as smoking and HPV infection were more common in subjects with lower socioeconomic status than higher one [20], [21].

The effectiveness of cervical cancer screening has been assessed over the past 30 years [22], [23], [24], [25]. In Korea, organized cervical cancer screening programs were introduced in 1988. Jun et al. [26] evaluated the effectiveness of the organized cervical cancer screening program in Korea and showed that regular cervical cancer screening reduced the risk of invasive cervical cancer (ICC) and CIS by 71% and 66%, respectively, in Korean women. However, only limited information is currently available on the performance of cervical cancer screening. As evaluation of performance indicators are the basis for the establishment of quality Korean cervical cancer screening program. This study provides detailed estimates of key outcome measures, including positivity rate, cancer detection rate and interval cancer rate (ICR). In addition, we determined the percentage of CIS among screen detected CIS+ (CIS or invasive cervical cancer) according to the socio-demographic factors.

## Methods

### Korean organized cervical cancer screening program

In 1988, the National Health Insurance Screening Program (NHISP) introduced an organized cervical cancer screening program, which provides free biennial Pap tests to National Health Insurance (NHI) beneficiaries aged 30 years or over. In 1999 the National Cancer Screening Program (NCSP) began offering the same screening opportunities to Medical Aid Program (MAP) recipients. Women in the target population receive an invitation letter from the NHI Corporation at the beginning of every other year.

Women invited voluntarily decide whether to make a screening appointment. They can do so at any clinic or hospital that is certified as a screening unit. During the screening visit they are asked to complete a cancer screening information sheet, and are subsequently screened. Results are usually sent to participants within 15 days. In individuals with a positive Pap test at screening, the organized cervical cancer screening guidelines recommend follow-up by either colposcopy or repeat Pap test. However, this follow-up test is not covered by the organized screening program, but by the NHI [27], [28].

### Study population

The present study was restricted to women aged 30 years or over who had undergone cervical cancer screening by Pap test through the NSCP or NHISP between January 1, 2005 and December 31, 2006. In total, 4,090,143 Pap test results were eligible for inclusion during this period. We then proceeded to exclude 607 (0.01%) missing screening results, 7,723 (0.19%) previous ICC diagnoses, and 8,816 (0.21%) previous CIS diagnoses, according to the Korean Central Cancer Registry (KCCR). Therefore the current analysis was based on 4,072,997 screenings.

The NCSP and NHISP databases included participants' demographic characteristics and screening results with written informed consents. We collected these data regularly from the NHI Corporation. As the NCSP and NHISP collect routine medical and health data, obtaining informed consent for this specific study was not necessary; this study was approved by the institutional review board of the National Cancer Center, Korea.

### Participant characteristics, Pap test assessment, and cancer diagnoses

Women provided information on socio-demographic characteristics, including age, health insurance status, health insurance premium per month and Residence Registration Number (RRN; a unique 13-digit number assigned to all Koreans). We used health insurance status (MAP vs. NHI) and NHI premium level as a proxy for socioeconomic status. These indicators have been regarded as a highly reliable proxy for real income [29]. Hence individuals were classified into three groups: MAP recipients (extremely poor people who received livelihood assistance and were unable to pay for health care or insurance); NHI beneficiaries with a premium under 50%; and NHI beneficiaries with a premium at 50% or above.

Pap tests (conventional cytology) were conducted by medical staff at local hospitals following a standardized procedure. Pap test results were reported using the Bethesda System, i.e., normal, infection/inflammation/reactive change, atypical squamous cells of undetermined significance, low-grade squamous intraepithelial lesion, high-grade squamous intraepithelial lesion, suggestive of squamous cell carcinoma, and other. Most Pap test results in the other category were glandular abnormalities (e.g., glandular atypia, atypical endocervical glands). A letter was sent to all participants with the overall test result, reported according to four categories: I, negative; II, benign; III, suspicious; IV, highly suggestive of malignancy. Results were defined as abnormal if an epithelial cell abnormality was reported and was coded as “suspicious” or “highly suggestive of malignancy” in the overall test results.

The final diagnosis was ascertained through linkage with the KCCR using the RRN, a nationwide hospital-based system that contains 95% of newly diagnosed malignancies in Korea [30]. In the KCCR, all malignant neoplasms and *in situ* cases are classified according to the International Classification of Diseases for Oncology, 3rd edition [31] and converted according to the International Classification of Diseases, 10th edition (ICD-10) [32]. We used the data on cancer diagnoses reported to the KCCR up to December 2007 to account for a 12-month period after screening, so that any follow-up or diagnostic work-up could be completed and the results fully reported. In the KCCR, cervical intraepithelial neoplasia is not registered; therefore, only cases of ICC (ICD-10: C53) and CIS (ICD-10: D06) were included in this study.

### Definitions and statistical analysis

We calculated the following performance measures: positivity rates for screening, detection rate, ICR, sensitivity, and specificity. The results of cervical cancer screening were categorized as negative (negative or benign disease of the cervix) or positive (suspicious or highly suggestive of malignancy). Screening assessment was based on Pap tests only.

The positivity rates (i.e., the proportion of screening assessments that led to a recommendation for further work-up such as colposcopy or biopsy to diagnose cancer) were calculated as the number of positive findings per 100 screenings. The detection rate was calculated as the number of cancer or CIS detected per 1,000 screenings. The ICR was estimated as the number of cancer diagnosed within 1 year of a negative screening per 1,000 negative screenings. Sensitivity was defined as the probability of a positive Pap test result given a finding of cancer within 1 year after screening [true positive/(true positive+false negative)]. Specificity was defined as the probability of a negative Pap test given no finding of cancer within 1 year after a screening [true negative/(true negative+false positive)].

Ninety-five percent confidence intervals (CI) were calculated for detection rate, ICR, sensitivity, specificity, and percentage of CIS+ that were CIS. The age distribution was significantly different by health insurance status (results not shown). Therefore, in multiple logistic regression, odds ratios (OR) for detection rate, ICR, and percentage of CIS among screen detected CIS+ were calculated after adjusting for age group and health insurance status. SAS software (version. 9.1; SAS Institute, Inc., Cary, NC, USA) was used for all statistical calculations.

## Results

### Screening results

A total of 4,072,997 cervical cancer screenings were performed from January 2005 to December 2006. About 74.3% of results were classified as negative, and 19.1% were classified as benign. The proportion of results classified as highly suggestive of malignancy was 0.9%. Subjects in their forties and MAP recipients had the highest percentage of results highly suggestive of malignancy. The positivity rate was 6.6%. Women in their thirties and NHI beneficiaries with a premium over 50% had the highest percentage of abnormal results (Table 1).

**Table 1:** Percent distribution of Pap test results by age group and health insurance status in the Korean organized cancer screening program, 2005–2006.

	No. of Screenings, n	Results, %				Positivity^a^, n (%)
		Negative	Benign	Suspicious	Highly suggestive of malignancy	
Total	4,072,997	74.31	19.09	5.66	0.94	268,845 (6.60)
Age group, y						
30–39	245,148	74.45	18.31	6.24	1.01	17,765 (7.25)
40–49	1,469,283	73.79	19.39	5.72	1.09	100,084 (6.81)
50–59	1,264,922	73.71	19.83	5.63	0.83	81,692 (6.46)
60+	1,093,644	75.65	18.01	5.47	0.87	69,304 (6.34)
Health insurance status with average monthly premium level						
MAP	206,672	75.15	18.74	4.85	1.26	12,626 (6.11)
NHI with premium under 50%	1,992,250	74.04	19.44	5.60	0.92	129,939 (6.52)
NHI with premium over 50%	1,874,064	74.50	18.76	5.80	0.93	126,278 (6.74)

MAP, Medical Aid Program; NHI, National Health Insurance.

a including suspicious and highly suggestive of malignancy.

### Performance of Pap test in cervical cancer screening

A total of 3,539 CIS+ were detected by Pap test (1,297 ICC and 2,242 CIS). The detection rates for ICC, CIS, and CIS+ were 0.32, 0.55, and 0.87 per 1,000 screens, respectively. The detection rate for ICC increased significantly with age (*P*_trend_<0.001), whereas the detection rate for CIS decreased significantly with age (*P*_trend_<0.001). In the logistic model, MAP recipients showed significantly higher ORs of detection rate for CIS+ (OR: 1.35, 95% CI: 1.17–1.55) and ICC (OR: 1.67, 95% CI: 1.35–2.06) than did NHI beneficiaries with a premium over 50% (Table 2).

**Table 2:** Detection rate (DR) of cervical carcinoma *in situ* or worse (CIS+) in screening participants aged 30 years and over, stratified by age group and health insurance status, the Korean organized cancer screening program, 2005–2006.

		CIS and ICC			ICC			CIS	
	No. detected	DR, per 1,000 (95% CI)	aOR (95% CI)^a^	No. detected	DR, per 1,000 (95% CI)	aOR (95% CI)^a^	No. detected	DR, per 1,000 (95% CI)	aOR (95% CI)^a^
Total	3,539	0.87 (0.84–0.90)		1,297	0.32 (0.30–0.34)		2,242	0.55 (0.53–0.57)	
Age group, y									
30–39	221	0.90 (0.78–1.02)	1.00	43	0.18 (0.12–0.23)	1.00	178	0.73 (0.62–0.83)	1.00
40–49	1,345	0.92 (0.87–0.96)	0.99 (0.85–1.14)	354	0.24 (0.22–0.27)	1.31 (0.96–1.80)	991	0.67 (0.63–0.72)	0.91 (0.77–1.07)
50–59	865	0.68 (0.64–0.73)	0.73 (0.63–0.85)	346	0.27 (0.24–0.30)	1.47 (1.07–2.02)	519	0.41 (0.38–0.45)	0.55 (0.46–0.65)
60+	1,108	1.01 (0.95–1.07)	1.07 (0.93–1.24)	554	0.51 (0.46–0.55)	2.68 (1.96–3.66)	554	0.51 (0.46–0.55)	0.68 (0.57–0.80)
*p-*value for trend			0.46			<0.001			<0.001
Health insurance status with average monthly premium level									
MAP	232	1.12 (0.98–1.27)	1.35 (1.17–1.55)	109	0.53 (0.43–0.63)	1.67 (1.35–2.06)	123	0.60 (0.49–0.70)	1.17 (0.97–1.41)
NHI(under 50%)	1,827	0.92 (0.88–0.96)	1.19 (1.11–1.27)	697	0.35 (0.32–0.38)	1.31 (1.17–1.48)	1,130	0.57 (0.53–0.60)	1.12 (1.03–1.22)
NHI(over 50%)	1,480	0.79 (0.75–0.83)	1.00	491	0.26 (0.24–0.29)	1.00	989	0.53 (0.49–0.56)	1.00
*p-*value for trend			<0.001			<0.001			0.006

aOR, adjusted odds ratio; CIS: carcinoma *in situ*; CI, confidence interval; ICC: invasive cervical cancer; MAP, Medical Aid Program; NHI, National Health Insurance.

a aOR is adjusted for variables in the table.

In total, 503 interval cancers occurred within 1 year of a negative screening result (ICR, 0.13/1000). The ICR increased significantly with age (*P*_trend_<0.001). The OR of ICR was 1.66 times higher for MAP recipients than it was for the NHI beneficiaries with premiums over 50% (Table 3).

**Table 3:** Interval cancer rates (ICR) of cervical cancer in screening participants aged 30 years and over, stratified by age group and health insurance status, the Korean organized cancer screening program, 2005–2006.

	No. of negative screening	No. of interval cancer	ICR^a^ (95% CI)	aOR (95% CI)^b^
Total	3,804,152	503	0.13 (0.12–0.14)	
Age group, y				
30–39	227,383	21	0.09 (0.05–0.13)	1.00
40–49	1,369,199	152	0.11 (0.09–0.13)	1.15 (0.73–1.82)
50–59	1,183,230	142	0.12 (0.10–0.14)	1.22 (0.77–1.94)
60+	1,024,340	188	0.18 (0.15–0.21)	1.84 (1.17–2.90)
*p-*value for trend				<0.001
Health insurance status with average monthly premium level				
MAP	194,046	40	0.21 (0.15–0.27)	1.66 (1.18–2.34)
NHI (under 50%)	1,862,311	271	0.15 (0.13–0.17)	1.31 (1.09–1.58)
NHI (over 50%)	1,747,786	192	0.11 (0.09–0.13)	1.00
*p-*value for trend				<0.001

aOR, Adjusted odds ratio; CI, confidence interval; MAP, Medical Aid Program; NHI, National Health Insurance.

a per 1,000 negative screenings.

b aOR is adjusted for variables in the table.

The sensitivity of Pap test to detect CIS (79.8%, 95% CI: 78.3–81.3) was higher than that for ICC (72.1%, 95% CI: 70.0–74.2%). Overall the sensitivity and specificity of Pap test to detecting CIS or ICC were 76.8% and 93.5%, respectively (Table 4).

**Table 4:** Sensitivity (sen) and specificity (spe) for detecting of cervical cancer by age group and health insurance status, the Korean organized cancer screening program, 2005–2006.

	CIS and ICC		ICC		CIS	
	Sen (95% CI)	Spe (95% CI)	Sen (95% CI)	Spe (95% CI)	Sen (95% CI)	Spe (95% CI)
Total	76.8 (75.6–78.0)	93.5 (93.5–93.5)	72.1 (70.0–74.2)	93.4 (93.4–93.4)	79.8 (78.3–81.3)	93.4 (93.4–93.4)
Age group, y						
30–39	76.2 (71.3–81.1)	92.8 (92.7–92.9)	67.2 (55.7–78.7)	92.8 (92.7–92.9)	78.8 (73.5–84.1)	92.8 (92.7–92.9)
40–49	75.4 (73.4–77.4)	93.3 (93.3–93.3)	70.0 (66.0–74.0)	93.2 (93.2–93.2)	77.5 (75.2–79.8)	93.2 (93.2–93.2)
50–59	75.7 (73.2–78.2)	93.6 (93.6–93.6)	70.9 (66.9–74.9)	93.6 (93.6–93.6)	79.2 (76.1–82.3)	93.6 (93.6–93.6)
60+	79.5 (77.4–81.6)	93.8 (93.8–93.8)	74.7 (71.6–77.8)	93.7 (93.7–93.7)	85.1 (82.4–87.8)	93.7 (93.7–93.7)
Health insurance status with average monthly premium level						
MAP	78.4 (73.7–83.1)	94.0 (93.9–94.1)	73.2 (66.1–80.3)	93.9 (93.8–94.0)	83.7 (77.7–89.7)	93.9 (93.8–94.0)
NHI (under 50%)	76.9 (75.2–78.6)	93.6 (93.6–93.6)	72.0 (69.2–74.8)	93.5 (93.5–93.5)	80.3 (78.2–82.4)	93.5 (93.5–93.5)
NHI (over 50%)	76.3 (74.4–78.2)	93.3 (93.3–93.3)	71.9 (68.5–75.3)	93.3 (93.3–93.3)	78.7 (76.4–81.0)	93.3 (93.3–93.3)

CIS: carcinoma *in situ*; CI, confidence interval; ICC: invasive cervical cancer; MAP, Medical Aid Program; NHI, National Health Insurance.

### Percentage of CIS among screen-detected CIS+

The percentages of CIS among screen-detected CIS+ varied with age and health insurance (Table 5). Among CIS+ diagnosed during the study period, 63.4% were CIS. The percentage of CIS decreased significantly with age (*P*_trend_<0.001). The likelihood of CIS was significantly lower for MAP recipients than for NHI beneficiaries with premiums over 50%. Among screen-detected CIS+, the percentage of CIS was 63.4% (95% CI: 61.8–65.0%), and this percentage was higher among women aged 30–39 years (80.5%, 95% CI: 75.3–85.7) than among those in all older age groups [ranging from 60.0% (95% CI: 56.7–63.3%) to 50.0% (95% CI: 47.1–52.9%) among women aged ≥50 years. *P*_trend_<0.001].

**Table 5:** Percentage of carcinoma *in situ* (CIS) cases among screen detected CIS+ by age and health insurance, the Korean organized cancer screening program, 2005–2006.

	No. of detected^a^	No. of CIS	% of CIS (95% CI)	aOR(95% CI)^b^
Total	3,539	2,242	63.4 (61.8–65.0)	
Age group, y				
30–39	221	178	80.5 (75.3–85.7)	1.00
40–49	1,345	991	73.7 (71.3–76.1)	0.84 (0.49–1.46)
50–59	865	519	60.0 (56.7–63.3)	0.44 (0.25–0.77)
60+	1,108	554	50.0 (47.1–52.9)	0.24 (0.13–0.42)
*p-*value for trend				<0.001
Health insurance status with average monthly premium level				
MAP	232	123	53.0 (46.6–59.4)	0.58 (0.33–1.01)
NHI (under 50%)	1,827	1,130	61.9 (59.7–64.1)	0.76 (0.59–0.99)
NHI (over 50%)	1,480	989	66.8 (64.4–69.2)	1.00
*p-*value for trend				0.011

aOR, adjusted odds ratio; CI, confidence interval; MAP, Medical Aid Program; NHI, National Health Insurance.

a Including CIS and invasive cervical cancer.

b aORs adjusted for variables in the table.

## Discussion

To our knowledge, this is the first study performed with detailed screening results of Pap test from a nationwide cancer screening program in Korea. The positivity rate for all Pap tests was 6.6%. The number of ICC detected was 1,297 (0.32 per 1,000 screens), and 503 interval cancer (0.13 per 1,000 negative screens) were diagnosed. The sensitivity and specificity of Pap test for ICC were 72.1% and 93.4%, respectively. Approximately 63% of screen-detected CIS+ turned out to be CIS.

In this study, the positivity rates decreased with age. In accordance with previous studies, the percentage of abnormal Pap tests results was highest at younger ages, which indicates that younger people are more likely to have multiple sexual partners and have higher rates of HPV infection [33].

ICC is usually preceded by a long phase of pre-invasive disease. Therefore, the goal of screening is to identify and remove significant precancerous lesions, in addition to prevention of mortality. Several reports regarding the occurrence of *in situ* lesions have been published based on data from population-based cancer registries [34], [35], [36]. These data, however, do not specifically report the incidence of screen-detected disease, as cancer registries do not routinely collect information on screening practices. Unscreened women are included in cancer registry statistics, but estimates of CIS incidence based on data from population-based cancer registries tend to underestimate the incidence of CIS among screened women, as CIS is primarily detected by Pap test during screening. Reports from individual screening programs and case-series have provided estimates of the percentage of CIS. However, there have been few reports of the occurrence of *in situ* lesions among women who participate in large organized screening programs [37], [38]. In the present study, the CIS detection rate was approximately 0.55 per 1,000 screenings, and this rate decreased with age. Our results suggest that one case of CIS is detected for approximately every 1,818 Pap tests performed. This rate varies by age, ranging from approximately 1 in every 1,370 Pap tests among women aged 30–39 years to 1 in every 2,439 Pap tests among women aged 50–59 years.

In general, cervical cancer incidence and mortality are the most appropriate end points for validating the effectiveness of the Pap test as a tool for screening. However, the use of these end points requires a long observation period and a large number of subjects to achieve adequate statistical power. For that reason, performance indicators were established to assess the effectiveness of cervical cancer screening [39], and key performance indicators include cancer detection rates, percentage of small cancers detected, and ICR [40]. These indicators are used as surrogate markers of the effectiveness of an organized cancer screening program; if the performance of an organized program is good, then a reduction in mortality might be expected. In breast cancer screening programs, ICR has been inversely associated with a reduction in mortality, and surveillance for interval cancer is widely used to monitor the performance of screening programs [41], [42]. Therefore, in the current study, we estimated the cancer detection rate and ICR. The detection rates of ICC and CIS were 0.32 and 0.55 per 1,000 screens, respectively; the ICR was 0.13 per 1,000 negative screens. Few studies were found that evaluated ICR based on data from population-based organized cervical cancer screening program. Furthermore, most of these studies assessed the negative screening history of women diagnosed with ICC [43], [44], [45], making it difficult to directly compare the ICRs of different studies due to differences in the study designs and settings.

Whereas the CDR and ICR increased with age, the CIS detection rate decreased with age (*P*_trend_<0.001). These trends were associated with the incidence of ICC and CIS. According to the KCCR, although the age-specific incidence of ICC increased with age, the age-specific incidence of CIS was highest among women in their forties, after which incidence decreased with age [3]. With regard to health insurance status, the ORs for the detection rate and ICR of ICC for MAP recipients were 1.67 and 1.66 times higher than those for NHI beneficiaries with premiums over 50%. The high prevalence of ICC in women with low socioeconomic status might affect the detection rate and ICR by health insurance status. Several studies have reported an inverse association between socioeconomic status (measured by indicators such as education, income, or health insurance status) and ICC [46], [47], [48].

Among CIS+ in the current study, MAP recipients were more likely to have a positive screening result diagnosed as ICC than CIS, compared with NHI beneficiaries with premiums over 50%. There are some possible explanations for the relatively high proportion of ICC in MAP recipients, for example they may have been less likely to adhere to the recommended screening interval considering the progression from pre-invasive lesions to ICC. In this study, we could not identify the history of cervical cancer screening among our study subjects. Previous studies have reported that socioeconomic status is highly significantly negatively associated with a longer screening interval [49], [50]. In addition, screening interval has been shown to be significantly positively associated with a cytological prediction of diagnosis [10], [49].

In this study, we found that the Pap test was more sensitive for the detection of CIS (79.8%, 95% CI: 78.3–81.3) than ICC (72.1%, 95% CI: 71.0–74.2). CIS after negative screening is much less common than interval ICC as symptoms such as irregular bleeding (e.g., bleeding between periods, with heavier or lighter amounts than normal menstrual flow, or bleeding following intercourse) might never become clinically apparent. Furthermore, compared with ICC, less CIS cases were found during opportunistic cervical cancer screening. This phenomenon may lead to an overestimation of the ability of Pap tests to detect *in situ* disease, and introduce bias in the sensitivity for detecting CIS.

The use of Pap test in cervical cancer screening has become widespread. Based on 2009 nationally representative data from the Korean National Cancer Screening Survey, 76.1% of Korean women aged ≥30 years had at least one Pap test in her lifetime, and 63.9% had one within the previous 2 years [51]. Our data suggest that approximately 1 in every 1,667 Pap tests results in a diagnosis of CIS. If early detection and treatment of CIS have contributed to the recent decline in cervical cancer mortality in Korea, independent of effects attributable to early detection and treatment of invasive disease and of recent advances in cervical cancer treatment, then some women who have been treated for screen-detected CIS have benefited.

A number of limitations should be considered when reviewing the results of the present study. They result primarily from the use of data collected as part of the organized screening program. First, screening program results were influenced by the policies used to administer the program. For example, we could not distinguish between symptomatic and asymptomatic participants, but individuals who reported symptoms were eligible for cervical cancer screening through the organized cancer screening program for ethical reasons, as they were more likely to have abnormal results and require diagnostic follow-up compared with asymptomatic participants. Second, we were unable to obtain records on Pap test history, which might affect the performance of screening. Third, although the organized cancer screening program is population-based, our results might not be generalizable because of a low participation rate. Overall, 19.8–22.4% of the invited women were screened. Furthermore, our study was unable to control self-selection bias because we did not have information about participant characteristics such as education level, employment status, and risk factors. In recent Korean study, participation in organized screening was relatively more concentrated among the lower-income groups [52]. Further study is needed to assess the performance of organized cancer screening linked with opportunistic screening. Fourth, the cervical cancer screening program was conducted as part of a medical examination, and data available to the NCSP and NHICSP do not include referral information (i.e., colposcopy or biopsy) or diagnostic test results. Finally, health insurance status and NHI premium level were used as a proxy for socioeconomic status in the present study. However, as we could not consider a participant's educational level or occupation as a proxy for socioeconomic status, it is possible that the socioeconomic status of participants was not fully reflected.

Despite these limitations, we believe that our study also has many strengths. To the best of our knowledge, this is the first study to present detailed results of cervical cancer screening from the nationwide screening program in Korea. In particular, we tried to investigate the distribution of CIS in a population-based Pap test program. The proportions of CIS were substantially different according to socio-demographic factors. In particular, the likelihood of a CIS diagnosis among CIS+ was significantly lower among MAP recipients than among NHI beneficiaries with higher income status. The difference in performance of cervical cancer screening among different socio-demographic groups may indicate an important influence of socio-demographic factors on preventive behavior. The findings of the study support the critical need for increasing efforts to raise awareness and provide more screening in at-risk populations, specifically low-income groups.

Evaluation of screening performance enables us to assess the interim effectiveness of cancer screening programs, to monitor the performance of the various components of the screening process, and to facilitate inter-jurisdictional comparisons. The evaluations of performance indicators are the basis for the establishment of standards for quality control. Further study is required to determine the acceptable level of several indicators for cancer screening policy and quality in Korea. Finally, establishment of standards and continuous performance monitoring according to socioeconomic status will help to reduce disparities in cervical cancer detection and care.
